# Supplementary material for: Proliferation and Cluster Analysis of Neurons and Glial Cell Organization on Nanocolumnar TiN Substrates
Source: Int J Mol Sci. 2020 Aug 28;21(17):6249. doi: 10.3390/ijms21176249 (PMC7503702; doi:10.3390/ijms21176249)
Supplement: Supplementary file 1 [file ijms-21-06249-s001.pdf]

Supplementary Materials

Proliferation and Cluster Analysis of Neurons and Glial Cell Organization on Nanocolumnar TiN Substrates

Alice Abend [1], Chelsie Steele [1], Sabine Schmidt [2], Ronny Frank [2], Heinz-Georg Jahnke [2,*] and Mareike Zink [1,*]

[1] Soft Matter Physics Division and Biotechnology & Biomedical Group, Peter-Debye-Institute for Soft Matter, Physics, Leipzig University, Linnéstr. 5, 04103 Leipzig, Germany; alice.abend@uni-leipzig.de (AA); cs73gygu@studserv.uni-leipzig.de (CS); zink@physik.uni-leipzig.de (MZ)

[2] Centre for Biotechnology and Biomedicine, Molecular Biological-biochemical Processing Technology, Leipzig University, Deutscher Platz 5, 04103 Leipzig, Germany; sabine.schmidt@bbz.uni-leipzig.de (SS); ronny.frank@bbz.uni-leipzig.de (RF); heinz-georg.jahnke@bbz.uni-leipzig.de (HGJ)

* Correspondence: zink@physik.uni-leipzig.de (MZ), heinz-georg.jahnke@bbz.uni-leipzig.de (HGJ); Tel.: +49 (341) 9732573, +49 (341) 9731246 (HGJ) (MZ); Fax: +49 (341) 9732479 (MZ), +49 (341) 9731249 (HGJ)

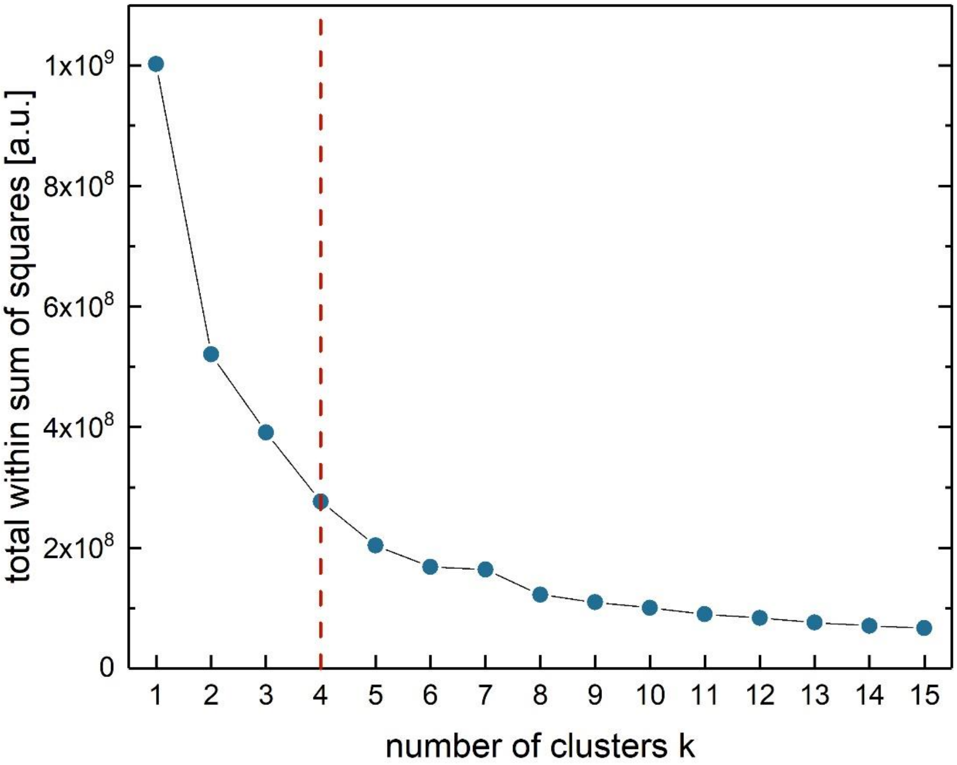

Figure S1: Result of the application of the elbow method to the data shown in Figures 5 and 6 (SH-SY5Y cells grown on TiN nanocolumnar substrates after 3 days). The red dashed line marks the optimal number of clusters k=4 for this example.

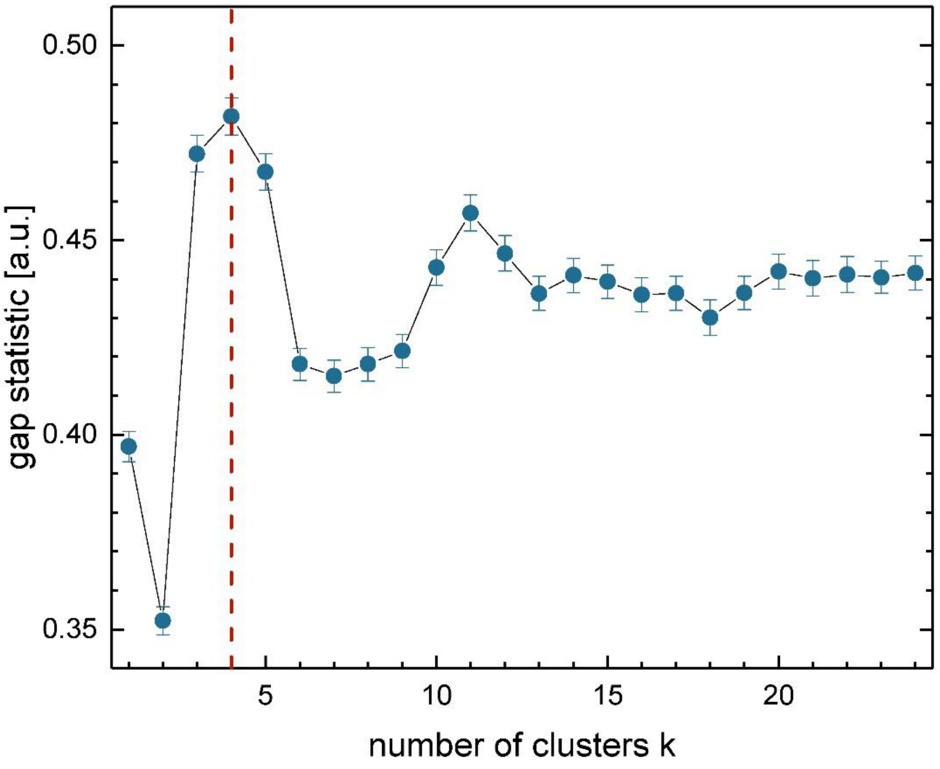

Figure S2: Result of gap statistics method applied to the data set shown in Figures 5 and 6 (SH-SY5Y cells grown on TiN nanocolumnar substrates after 3 days). The value $Gap(k)$ was calculated according to Equation 3. The optimal number of clusters (red dashed line) is found to be k=4 in this case indicated by the peak of the graph. Most of our gap statistics graphs show this distinct maximum and thus reveal a reliable result for the optimal number of clusters. The gap statistics curve could theoretically also exhibit a slowly ascending and then flattening form so that the condition $Gap(k) \geq Gap(k+1)$ is never fulfilled. Therefore, this condition is extended by the standard deviation term in Equation 4. The condition $Gap(k) \geq Gap(k+1) - s(k+1)$ can usually be fulfilled somewhere in the graph.

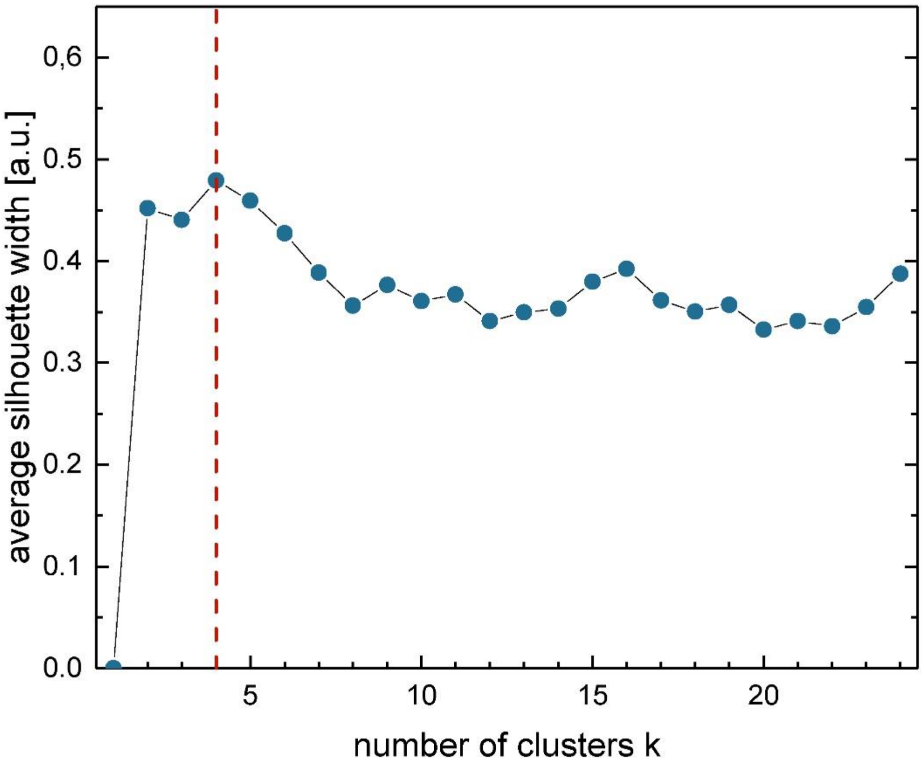

Figure S3: Average silhouette width associated with the example from Figures 5 and 6 (SH-SY5Y cells grown on TiN nanocolumnar substrates after 3 days). The maximum of the curve represents the optimal number of clusters for this experiment (red dashed line). We therewith verify our results drawn from the elbow method and gap statistics (see Figures S1 and S2).
